# Characterization, Microbial Community Structure, and Pathogen Occurrence in Urban Faucet Biofilms in South China

**DOI:** 10.1155/2015/401672

**Published:** 2015-07-26

**Authors:** Huirong Lin, Shuting Zhang, Song Gong, Shenghua Zhang, Xin Yu

**Affiliations:** Institute of Urban Environment, Chinese Academy of Science, Xiamen 361021, China

## Abstract

The composition and microbial community structure of the drinking water system biofilms were investigated using microstructure analysis and 454 pyrosequencing technique in Xiamen city, southeast of China. SEM (scanning electron microscope) results showed different features of biofilm morphology in different fields of PVC pipe. Extracellular matrix material and sparse populations of bacteria (mainly rod-shaped and coccoid) were observed. CLSM (confocal laser scanning microscope) revealed different distributions of attached cells, extracellular proteins, *α*-polysaccharides, and *β*-polysaccharides. The biofilms had complex bacterial compositions. Differences in bacteria diversity and composition from different tap materials and ages were observed. Proteobacteria was the common and predominant group in all biofilms samples. Some potential pathogens (Legionellales, Enterobacteriales, Chromatiales, and Pseudomonadales) and corrosive microorganisms were also found in the biofilms. This study provides the information of characterization and visualization of the drinking water biofilms matrix, as well as the microbial community structure and opportunistic pathogens occurrence.

## 1. Introduction

During the past decades, scientists have paid great attention to the biofilms formation in drinking water distribution systems (DWDSs) since biofilms formation and its resistance to disinfection were considered to be potential risk in DWDSs [1–3]. Bacteria can grow in bulk water and become attached to pipe walls as biofilms. Biofilms formation in DWDSs can lead to public health issues, such as protecting pathogenic bacterial regrowth, and depletion of disinfectant [4–6]. The microbial quality of tap water is closely related to consumers' health. Thus, knowledge of biofilms behavior in DWDSs and faucet can contribute to the design of effective control strategies.

DWDSs are engineered environments that are subject to frequent, variable disturbances caused by many factors including long residence times, each associated with loss of disinfectant residual and generating higher levels of biofilms growth. These changes are reflected in the new phenotypic characteristics developed by biofilm bacteria and occurred in response to a variety of environmental signals. Phylogenetically diverse bacterial groups can inhabit the biofilms attached to the pipes and the bulk water. The study of bacterial ecology can improve the understanding of the persistence of biofilms and pathogens.

Studies to date suggest that the planktonic-biofilm transition is a complex and highly regulated process. Several methods including culture-based and independent analyses showed that members of the class Proteobacteria, including the Alpha-, Beta-, and Gammaproteobacteria were considered to be the most predominant bacterial groups in water distribution systems [7]. Recent genetic and molecular approaches used to study bacterial and fungal biofilms have identified genes and regulatory circuits important for initial cell-surface interactions, biofilm maturation, and the return of biofilm microorganisms to a planktonic mode of growth [1, 3, 7]. However, the characterization of microbial communities of biofilms in DWDSs is far from being fully understood. To give insight into the characterization and bacterial diversity of biofilms in drinking water distribution systems, microstructure analysis (SEM, CLSM and XRF) was used for the characterization of biofilms on faucet. The bacterial community compositions of real biofilms from 12 urban household taps (Xiamen, southeast of China) were detected using 454 pyrosequencing technique. Potential pathogens and corrosive microorganisms occurrence was also discussed.

## 2. Methods

### 2.1. Characterization and Visualization of the Biofilms Matrix in DWDSs

In order to observe the biofilms formed on drinking water pipe surface, five 5 × 5 mm removable PVC sheets were inserted in real PVC DWDSs pipe for biofilm attachment. They were sterilized by immersion in 70% ethanol for 2 min individually and air dried. The PVC tap in our lab was twisted off and the sheets were transferred with sterile forceps to the tap; then, the tap was screwed on. After three months, PVC sheets were took out for scanning electron microscopy (SEM) and staining. One PVC sheet was fixed, dried, and viewed with a scanning electron microscope (Hitachi S-4800, Japan). The other four were stained with DAPI (4,6-diamidino-2-phenylindole) for attach cells, FITC (fluorescein isothiocyanate) for extracellular protein, Con-A (concanavalin A) for *α*-polysaccharide, and CW (calcofluor white) for *β*-polysaccharide, respectively. Each kind of stain was added on each sheet in the dark. After 15 min the sheets were analyzed using CLSM. CLSM 3D-image was used to visualize the staining biofilms. The CLSM scanned different layers of the staining biofilms. The thickness of the biofilms was got from signals of different layers using the software ZEN 2008 Light Edition.

### 2.2. Biofilms Sampling for 454 Pyrosequencing

It is difficult to get the information on the microbial diversity of biofilms within DWDSs due to limited access and the high cost involved in sampling. In this study, biofilms in taps were collected from a drinking water distribution system of Xiamen, Fujian Province in southeast of China. Different taps with different materials and different ages were studied (Table 1). The taps were removed and ethanol-sterilized cotton swabs were then used to collect biofilms on the taps. Then, the collected biofilms were moved to 2 mL sterilized EP tubes and put into an ice box during transportation. For each sampling point, the physicochemical water quality parameters were measured and listed in Table 1.

### 2.3. X-Ray Fluorescence (XRF) Analysis of Biofilms A3 on Corroded Tap

Among the biofilms samples, A3 was red, indicating that the pipe was corroded. In order to analyze the element contents in the biofilms, XRF analysis of the biofilms A3 was performed at the XRF microprobe station (beam line 4W1B) of Beijing Synchrotron Radiation Facility (BSRF), Institute of High Energy Physics of China. The electron energy in the storage ring was 2.2 GeV, with a current range from 78 to 120 mA. The size of the exciting X-ray beam was 10 *μ*m × 20 *μ*m. XRF spectra were collected by a PGY Si (Li) solid detector, positioned at 90° to the beam line. The scanning point of the sample was selected and observed by a microscope.

### 2.4. DNA Extraction, 16S rRNA Genes Amplification, and Pyrosequencing

Total DNA of the biofilms samples was extracted and purified using a bead beating method (FastDNATMSPIN Kit for Soil, Bio101 Inc., USA) following the manufacturer's instructions. For pyrosequencing analysis, the V3 and V4 region of 16S rRNA gene was amplified with the primers 338F (5′-ACTCCTACGGGAGGCAGCAG-3′) and 802R (5′-TACNVGGGTATCTAATCC-3′). The PCR was carried out in a total volume of 20 *μ*L: H_2_O 13.25 *μ*L, 10xPCR ExTaq Buffer 2.0 *μ*L, DNA template (100 ng/*μ*L) 0.5 *μ*L, primer 338F (10 mmol/L) 1.0 *μ*L, 802R (10 mmol/L) 1.0 *μ*L, dNTP 2.0 *μ*L, and ExTaq (5 U/mL) 0.25 *μ*L. The DNA amplification condition used in this study was initial denaturation at 95°C for 3 min, followed by 35 cycles of 10 s at 95°C, 30 s at 58°C, and 6 sec at 72°C, followed by a final extension at 72°C for 7 min. The PCR products were purified and end-repaired, A-tailed, PE-adapter ligated, and then utilized for pyrosequencing on the 454 Genome Sequencer FLX platform [8].

The unique tags (nonredundant tags) were obtained and aligned against the 16S rRNA database using the BLASTN algorithm. The raw sequences obtained from 454 pyrosequencing were optimized. The redundant tags were deleted by Mothur v.1.11.0 [9]. Sequences with similarities greater than 97% were grouped in one OTU. Using the MEGA5.0 program, the 60 bp representative sequences were aligned by the ClustalW with default settings, and a phylogenetic analysis was performed based on the neighbor-joining method [9].

## 3. Results and Discussion

### 3.1. Characterization and Visualization of the Drinking Water Biofilms Matrix

**Figure 1:**
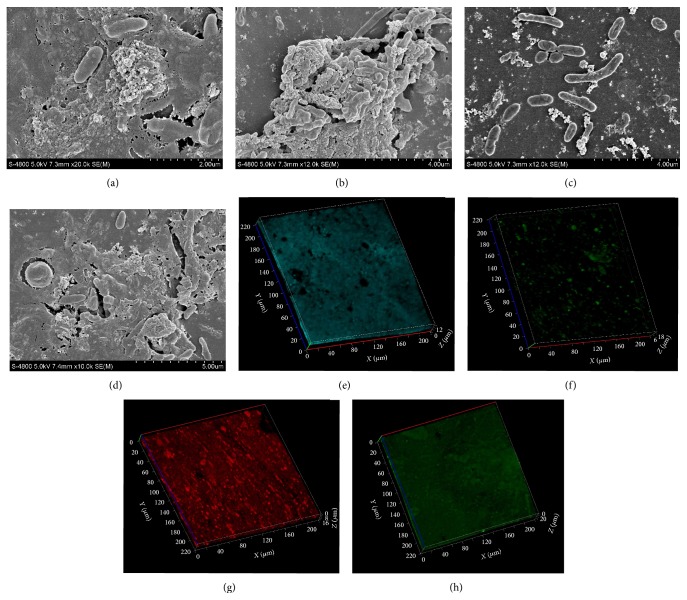
Visualization of the drinking water biofilms matrix on the PVC slice after three months growth. SEM images of extracellular matrix material (a, b) and rod-shaped bacteria (c) and globular-shaped bacteria (d), CLSM 3D-image of staining biofilms on the surface of the PVC slice after three months growth, DAPI for attach cells (e), FITC for extracellular protein (f), Con-A for *α*-polysaccharide (g), and CW for *β*-polysaccharide (h).

DWDSs are engineered environments which are subject to frequent, variable disturbances caused by many factors including long residence times due to dead ends and low flow rates, and the latter are associated with loss of disinfectant residual and generating higher levels of biofilm growth. Attachment is required for biofilms formation, and bacteria interact with pipe wall surface through adhesions including polysaccharides and surface proteins, with initial contact often mediated by active motility. The formation of a biofilm is the result of successional development into a mature community, which may require several years before steady state is achieved. For example, it was found that the successional development of a model DW biofilm during a 3-year period was an orderly process resulting in a stable, well-defined community [10]. In order to observe and characterize a real DWDSs biofilm, CLSM and SEM were applied in this study. SEM analysis of the PVC slice revealed different features of biofilms morphology in different fields of the PVC pipe surface. Possible EPS was present on the PVC slice in one field (Figures 1(a) and 1(b)). Sparse populations of mainly rod-shaped bacteria were observed in another field (Figure 1(c)). There were also globular-shaped bacteria (Figure 1(d)). Using Zen 2008 software, the “range” value showed the thickness of the biofilm. CLSM 3D-image results suggested that after three months' growth the biofilm was about 20 *μ*m thick. CLSM results also revealed different distributions of attached cells (Figure 1(e)), extracellular protein (Figure 1(f)), *α*-polysaccharide (Figure 1(g)), and *β*-polysaccharide (Figure 1(h)).

**Figure 2:**
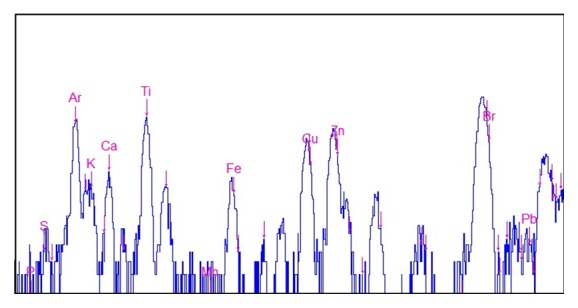
Typical XRF spectra in biofilm A3 from drinking water distribution stainless steel pipe wall. The Ar peak was from the air, while Ti and Br peak were artefact caused by 3M tape for fixing sample.

During sampling, it was found that there were some yellow iron filings in the biofilm A3, suggesting that the Fe in the stainless steel pipe was released. In order to obtain the elements contents of corroded biofilm A3, XRF was used.  Figure 2 was the typical spot scan result of biofilm A3. It showed that K, Ca, Fe, Cu, and Zn were present in biofilm A3, suggesting that this pipe was corroded and metal elements were released which might affect the water quality in the DWDSs. Our study enabled the concurrent visualization and evaluation of the biofilm growth as well as EPS matrix (extracellular proteins and polysaccharide).

### 3.2. Diversity of Microbial Communities in the Tap Biofilms

Pyrosequencing is a powerful tool to investigate microorganisms community [11]. Compared with conventional cloning and sequencing methods, pyrosequencing can obtain more sequences and OTUs. In this study, 8 biofilms samples were successfully high-throughput pyrosequenced among the 12 samples. To assess the sequencing depth and the species richness, a rare faction curve was constructed for each sample. Alpha diversity measurement for the samples (Table 2) suggested variations in species richness (Chao1) and species evenness (Simpson index) among different biofilms samples. Higher species richness of a given sample might present a lower species diversity resulting from a lower evenness of the sample. PcoA results indicated that the biofilms samples obtained from different tap materials showed quite different clusters. Biofilms formed on PVC had a more simple microbial community compared with those formed on cast iron and stainless steel. This result was consistent with another study which conducted with 16S rRNA genes sequence [12]. These results indicated that PVC was a more ideal material compared with the other materials as fewer bacteria attached on it.

### 3.3. Composition of Microbial Community

**Figure 3:**
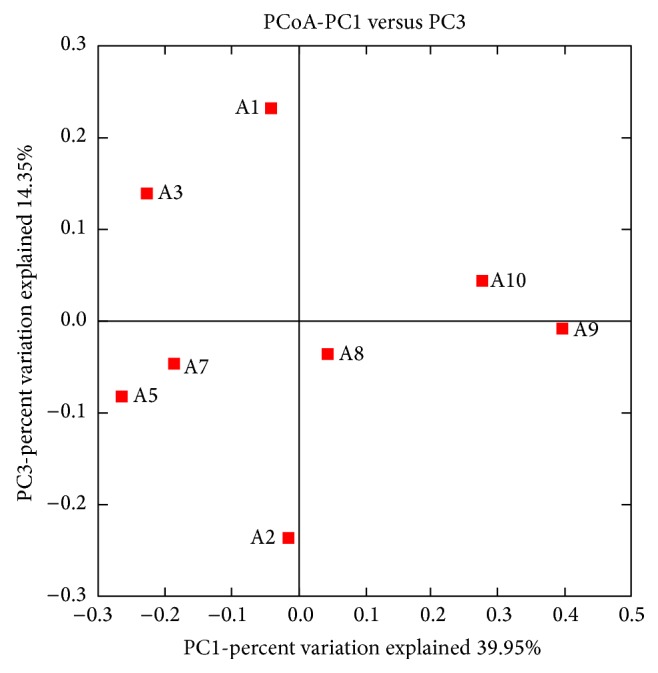
Phylogenetic analysis based on the 16S rRNA V3-V4 region sequences from the biofilms samples.

**Figure 4:**
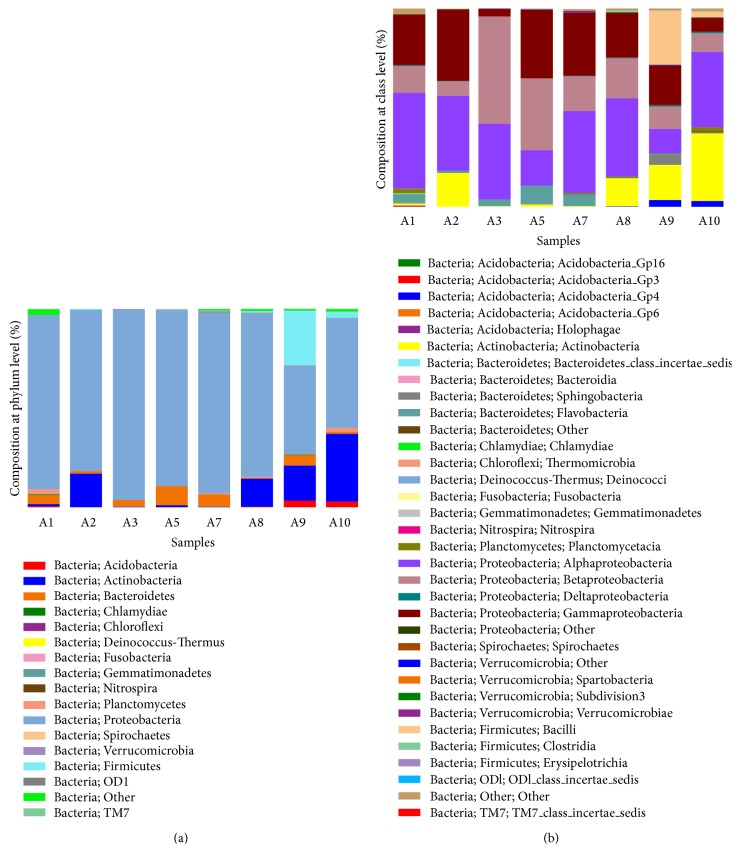
Proportional composition of microbes in the biofilms samples on different materials and from different ages at the phylum level (a) and class level (b). The information of each biofilm sample was listed in Table 1.

DWDSs biofilms are considered to be complex, dynamic microbial assemblages with extensive phenotypic diversity supporting adaptation to different hydraulic and chemical surface conditions [3, 13–15]. As a result, biofilms formed on different materials might have different community structures. The phylogenetic analysis based on the 16S rRNA V3-V4 region sequences from the biofilms samples was showed in Figure 3. The points in this figure were dispersive. It suggested that distinct bacterial communities were detected in these biofilms from different tap materials and different ages. Many factors could influence the bacterial communities including pipe age and pipe material. However, some interesting information could be found. For example, A8 and A9 (biofilms formed on cast iron) located in one region, while A1 and A3 (biofilms formed on stainless steel) located in another region. This result suggested that the pipe materials might be an important factor for the bacterial communities of the biofilms. In addition, biofilms A2 formed on PVC had relatively simple bacterial community structure, with Betaproteobacteria (54.09%) and Alphaproteobacteria (38.24%) as its main community composition (Figure 4). It was quite different from the biofilms formed on cast iron and stainless steel.

The bacterial communities of the samples in both class and species were shown in Figure 4. A high variability of the bacterial core communities of the biofilms was found in this study. Taxonomic analysis revealed that proteobacteria was the common and predominant group in all biofilms samples. These results were broadly consistent with other biofilms studies [7, 16]. Besides, Actinobacteria was the second largest bacterial group in the samples A2, A8, A9, and A10. Some important bacteria should be paid attention to. For example, Sphingomonadales was found in all samples. This might be due to the resistance to chlorine of Sphingomonadales. In addition, Nitrospiraceae and Thiotrichales were found in this study, suggesting the occurrence of nitrification and sulfur transformation.

### 3.4. Potential Pathogen and Corrosive Microorganisms Occurrence

Opportunistic pathogens in drinking water systems are an emerging health concern. In this study, some waterborne opportunistic pathogens were found in the biofilms samples. They were members of species Legionellales, Enterobacteriales, Chromatiales, Pseudomonadales, and Enterococcaceae, although there were differences in numbers for each sample. Abundance of some potential corrosive microorganisms and pathogens in genus level in different bacterial communities was listed in Table 3. These pathogen bacteria in the biofilms may be the risk to human health and lead to clinical and outbreak cases. In previous studies, it was found that environmental pathogens such as* Legionella pneumophila* and* Mycobacterium avium* have been shown to be associated with DW biofilms [1, 17, 18]. Our taxonomic findings are consistent with these studies. In some studies, it was found that Mycobacteria were found to be the predominant bacterial genera in DWDSs because they were generally resistant to disinfectants due to their complex cell wall [3, 18–20]. However, it was not detected in this study.

As corrosion was found in the biofilm A3 and XRF result showed high Fe, Cu, and Pb contents in the biofilms sample, the bacterial community information was further analyzed to understand the possible roles of microorganisms in iron corrosion. Thiotrichales (sulfur oxiding bacteria) was found in A1 biofilm sample form on stainless steel. In addition, potential iron reducing bacteria (Bacillales, Clostridiales, and Pseudomonadales) were found in some samples (Table 3). It was reported that IRB might generate Fe_3_O_4_ in anaerobic conditions, leading to corrosion in DWDSs. As a key impact factor to corrosion, the sulfate contents in the drinking water of this city are usually low according to the general survey. For example, survey data in 2012 showed that there were among the 102 samples, only 2 samples had the sulfate contents higher than 30 mg/L. So in this study, fewer corrosive microorganisms were found in this city, compared with another study which was conducted on the biofilms with higher sulfate contents in water [21]. Nevertheless, these corrosive microorganisms might contribute to corrosion of drinking water pipes. It should be noticed that the genus members within the genera* Bacillus* and* Clostridium* could affect iron corrosion and modify the corrosion products [22].

## 4. Conclusions

In this study, combined use of SEM, CLSM, and XRF was successfully conducted for the characterization and visualization of the drinking water biofilms matrix. EPS and bacteria (mainly rod-shaped and coccoid) were observed in the biofilms. Four hundred fifty-four pyrosequencing used to characterize bacterial diversity in these biofilms samples suggested that there were differences in the bacterial community composition between different biofilms samples formed on different tap materials with different ages. Proteobacteria (mostly Alpha-, Beta-, and Gamma-) predominated in the biofilms samples. Some potential pathogens (members of species Legionellales, Enterobacteriales, Chromatiales, and Pseudomonadales) were found in the biofilms samples. There were also some potential corrosive microorganisms (Thiotrichales, Bacillales, Clostridiales, and Pseudomonadales). In order to prevent biofilms formation, PVC might be a more ideal material compared with the others. This work might add some new insights into microbial community and its influential factors in DWDSs.

## Figures and Tables

**Table 1:** The biofilms samples used in this study and water quality parameters at each sampling location.

Samples	A1	A2	A3	A4	A5	A6	A7	A8	A9	A10	A11	A12
Tap material	Stainless steel	PVC	Stainless steel	Cast iron	Cast iron	Stainless steel	Stainless steel	Cast iron	Cast iron	Stainless steel	PVC	PVC
Age (year)	2	1	4	9	8	3	5	12	15	6	1	0.5
pH	7.08	6.8	7.01	6.62	7.11	7.03	6.94	7.35	7.26	6.83	6.98	6.7
TSS	40	49	99	75	81	69	39	80	69	73	52	47
Turbidity	0.6	0.3	0.3	0.2	0.4	0.2	0.1	0.3	0.2	0.1	0.4	0.3
Residual chlorine (mg/L Cl_2_)	0.55	0.54	0.62	0.46	0.49	0.63	0.65	0.57	0.61	0.62	0.3	0.4
Sulfate (mg/L)	10.4	12.9	31.8	24.2	30.6	13.8	24.2	22	27.8	19.8	17.8	16.5

**Table 2:** Comparison of phylotype coverage and diversity estimators of the microbial community structure employing the 454 pyrosequencing analysis.

Tap material	Samples	Reads	OTUs	ACE	Chao 1	Shannon	Simpson
PVC	A2	10,804	81	129	139	3.64	0.862

Stainless steel	A1	9,826	186	298	292	4.63	0.898
	A3	36,655	146	294	276	3.93	0.888
	A7	14,605	114	204	202	4.08	0.896
	A10	9,826	351	478	481	5.37	0.927

Cast iron	A5	13,660	106	182	192	3.27	0.831
	A8	13,214	217	383	340	4.43	0.921
	A9	27,517	152	250	256	4.46	0.916

**Table 3:** Abundance of some potential pathogen and corrosive microorganisms in genus level in different bacterial communities (count).

	genera	PVC	stainless steel				cast iron		
		A2	A1	A3	A7	A10	A5	A8	A9
Pathogen	*Escherichia_Shigella *	1	0	1	0	0	0	1	0
	*Legionella *	0	4	5	0	7	0	4	4
	*Pseudomonas *	13	27	70	39	444	66	429	1219
	*Enterococcus *	0	0	0	0	0	0	2	0

SOB	*Thiobacillus *	0	7	0	0	0	0	0	0

IRB	*Bacillus *	0	0	2	1	382	0	1	1826
	*Clostridium_XlVa *	0	0	0	0	0	4	0	0
	*Clostridium_XVIII *	0	0	0	0	0	0	1	0
	*Pseudomonas *	13	27	70	39	444	66	429	1219

SOB: sulfur oxidizing bacteria; IRB: iron reducing bacteria.
